# Supplementary material for: Surveillance of SARS-CoV-2 lineage B.1.1.7 in Slovakia using a novel, multiplexed RT-qPCR assay
Source: Sci Rep. 2021 Oct 14;11:20494. doi: 10.1038/s41598-021-99661-7 (PMC8516907; doi:10.1038/s41598-021-99661-7)
Supplement: Supplementary file 1 — Supplementary Information. [file 41598_2021_99661_MOESM1_ESM.pdf]

Supplementary information

Surveillance of SARS-CoV-2 lineage B.1.1.7 in Slovakia using a novel, multiplexed RT-qPCR assay

Kristína Boršová[3,4†], Evan D Paul[1,2†], Viera Kováčová[1,2†], Monika Radvánszka[1,2], Roman Hajdu[1,2,21], Viktória Čabanová[3], Monika Sláviková[3], Martina Ličková[3], Ľubomíra Lukáčiková[3], Andrej Belák[5,6], Lucia Roussier[6], Michaela Kostičová[6,7], Anna Líšková[8], Lucia Maďarová[9], Mária Štefkovičová[10,11], Lenka Reizigová[10,12], Elena Nováková[13], Peter Sabaka[14], Alena Koščálová[14,15], Broňa Brejová[16], Edita Staroňová[17], Matej Mišík[18], Tomáš Vinař[19], Jozef Nosek[20], Pavol Čekan[1,2,*], Boris Klempa[3,*]

Supplementary Figures

Supplementary Figure S1.

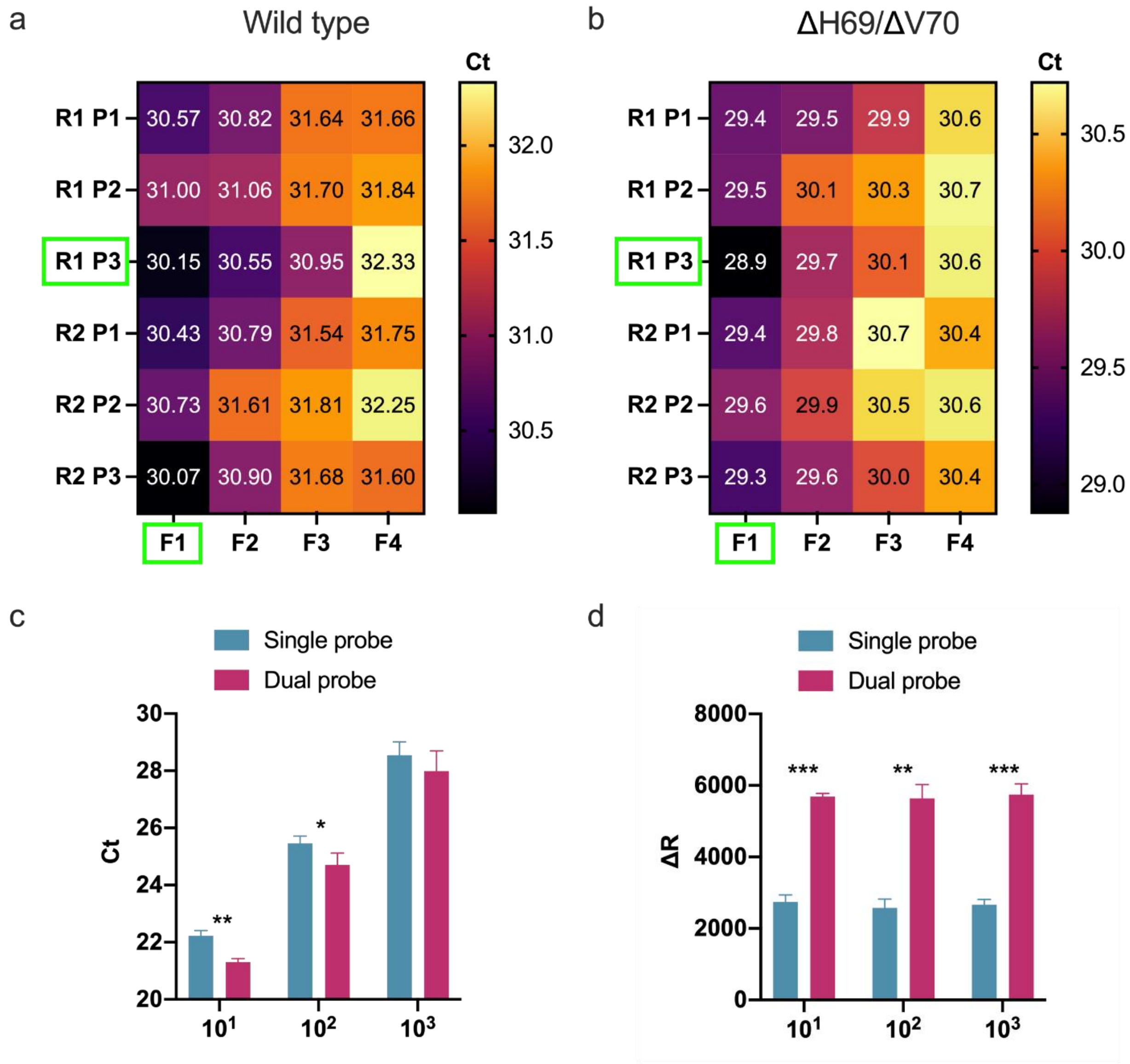

Supplementary Figure S1. Development and optimization of a general SARS-CoV-2 S gene primer/probe set for all SARS-CoV-2 variants.

a, b) Heatmaps illustrate oligonucleotide primer and probe combinations designed to target conserved sequences within the spike gene, including all SARS-CoV-2 variants that were contained in our bioinformatics analysis. Combinations of forward (F1-F4) and reverse primers (R1-R2) and hydrolysis probes (P1-P2) were tested using two separate SARS-CoV-2 variants, a common SARS-CoV-2 variant (Wild type, panel **a**) and a variant containing the ΔH69/ΔV70 deletion (**b**). Green rectangle boxes indicate best performing primer/probe combinations. **c, d)** Bar graphs compare RT-qPCR performance of a single probe versus an additional identically labelled dual probe using three 10-fold (10^1, 10^2, 10^3) dilutions of SARS-CoV-2 template ran in triplicates. Evaluation of the performance was done by comparing raw Ct values (**c**) and fluorescence intensity values (**d**). Statistical analysis was performed using paired t-test (***$p \leq 0.001$, **$p \leq 0.01$, *$p \leq 0.05$).

Supplementary Figure S2.

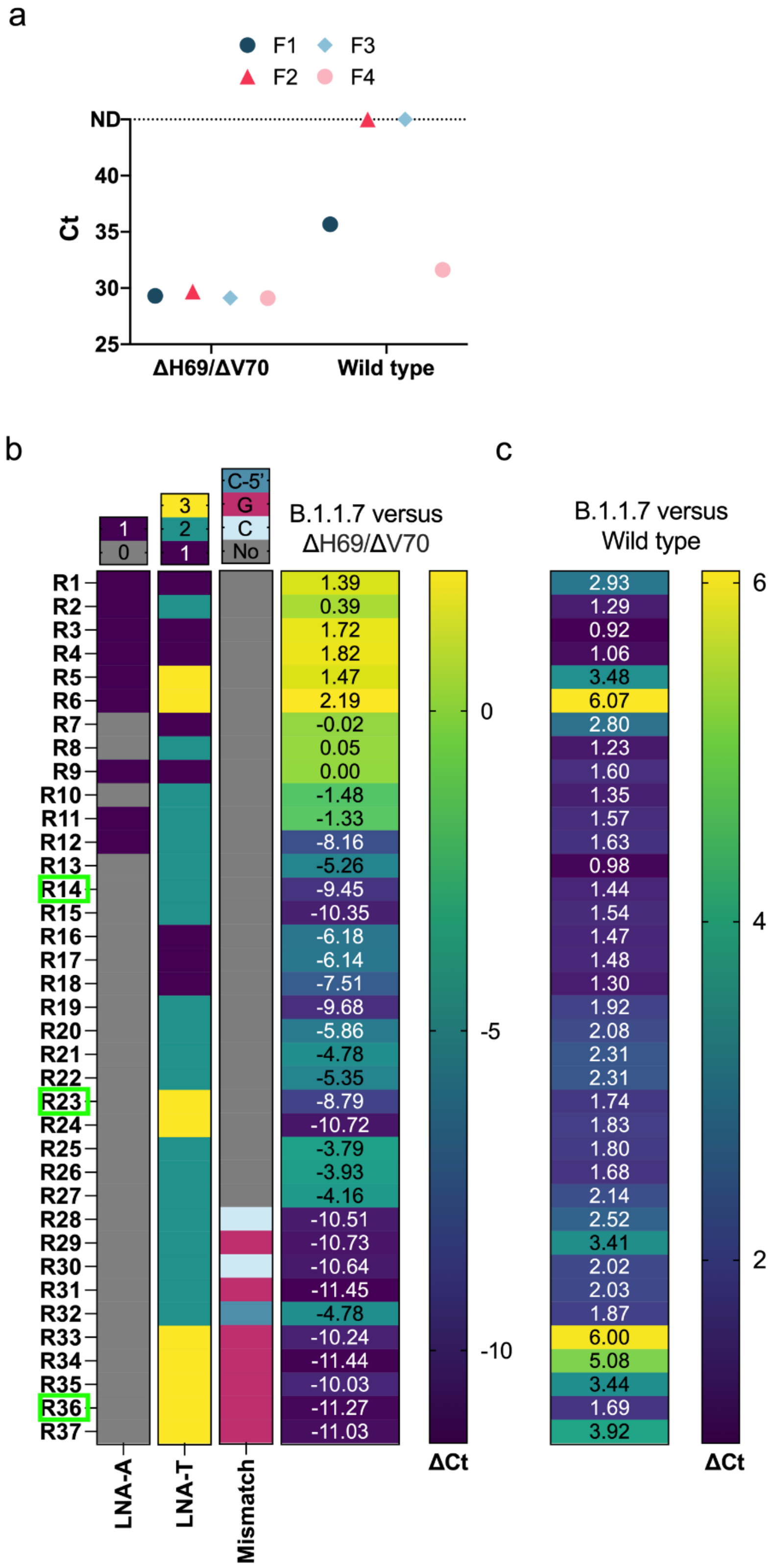

Supplementary Figure S2. Development and optimization of a spike gene primer/probe set specific for the B.1.1.7 SARS-CoV-2 variant.

a) Assessment of forward primers (F1-4) targeting the ΔH69/ΔV70 deletion in B.1.1.7 using the best reverse primer and probe (from **Supplementary Fig. S1**/ **Supplementary Table S2**). Symbols compare Ct values of the ΔH69/ΔV70 variant and wild type templates. Dotted line indicates samples that were not detected (ND) within 45 cycles. **b)** Overview of reverse primer designs targeting the ΔY144 deletion of the B.1.1.7 variant and their effects on specificity by comparing the relative ΔCt when amplifying either B.1.1.7 or ΔH69/ΔV70 variants as template. Darker colours in the heatmap represent a greater ΔCt and consequently better specificity. Green rectangle boxes indicate reverse primers selected for further optimization. LNA-A depicts primers containing an LNA modified adenine base located at either the 3'- or 5'-end of the reverse primer. LNA-T displays the number (1-3) of LNA-modified thymine bases for each reverse primer. Mismatch base represents design modifications to introduce either a guanine (G) or cytosine (C) mismatch base in either the penultimate base (G or C) or the 3rd from last base (C-5') relative to the 3'-end of the reverse primer. **c)** Heatmap shows ΔCt value comparison of B.1.1.7 primer/probe set to SARS-CoV-2 S gene primer/probe set using the B.1.1.7 variant as template. Darker colours indicate smaller ΔCt and consequently better specificity.

Supplementary Figure S3.

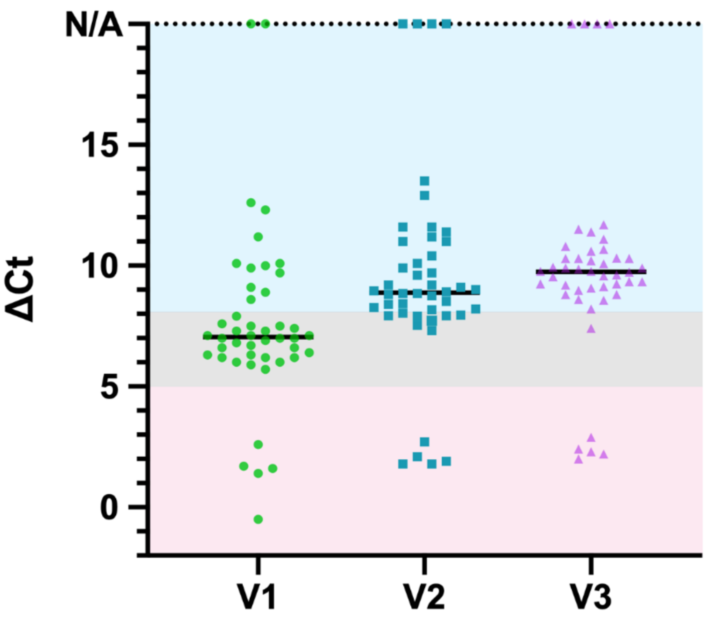

Supplementary Figure S3. Overview of B.1.1.7 assay performance on clinical samples. Three different versions (V1, V2, V3) of B.1.1.7 primer/probe sets that varied according to the reverse primer (V1, V2, and V3 use reverse primers R14, R23, and R36, respectively) were directly compared on a selected panel of 46 SARS-CoV-2 positive clinical samples, some of which were confirmed B.1.1.7 and B.1.258Δ variants by sequencing. ΔCt values correspond to B.1.1.7 assay Ct − SARS-CoV-2 S gene assay Ct. Coloured boxes within the plot define boundaries for corresponding variant interpretation, red (ΔCt ±5) for B.1.1.7, blue (ΔCt 8-20) for ΔH69/ΔV70, grey (ΔCt 5-8) for inconclusive samples. N/A represents samples which were detected only in SARS-CoV-2 S gene assay and therefore are interpreted as consensus SARS-CoV-2.

Supplementary Figure S4.

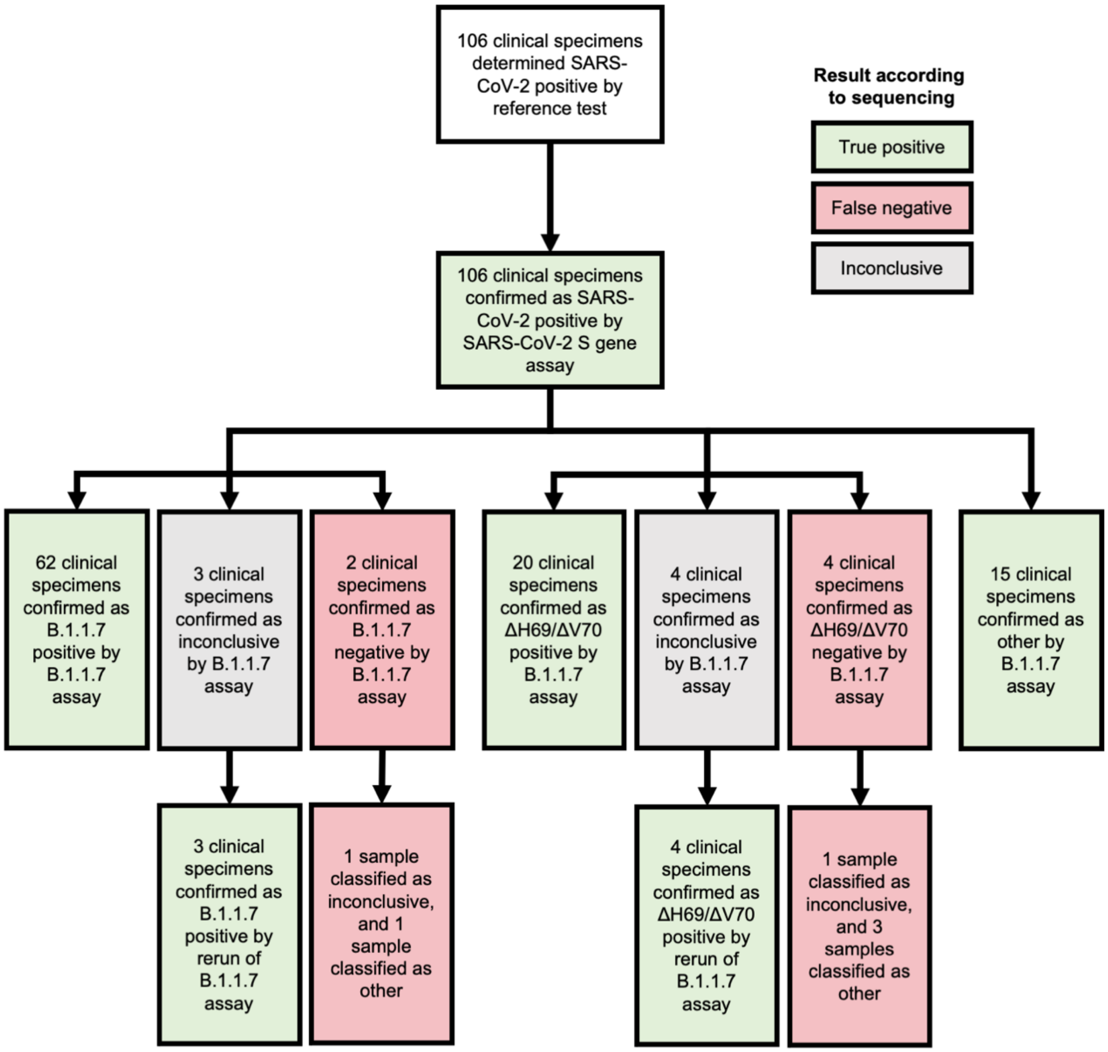

Supplementary Figure S4. Flow chart illustrating the patient samples used in the clinical evaluation

Samples (n=106) were considered valid if they were determined to be SARS-CoV-2 positive by a reference test used in routine testing in the Slovak Republic. These 106 samples were tested with both a SARS-CoV-2 S gene RT-qPCR, which detects all known SARS-CoV-2 variants, and a B.1.1.7 RT-qPCR assay that can differentiate variants that contain both the ΔH69/ΔV70 and ΔY144 deletions (e.g., B.1.1.7) from those that contain only the ΔH69/ΔV70 deletion (e.g., B.1.258). Seven samples did not meet the pre-specified criterion (**Supplementary Table S4**) and were deemed as inconclusive. These samples were subjected to a repeat test based on the pre-specified instructions (**Supplementary Table S4**). The colored boxes denote the concordance of the B.1.1.7 RT-qPCR results with the lineage status determined by genome sequencing.

Supplementary Tables

Supplementary Table S1. Origin and genomic characterization of GISAID sequences used for alignment and primer/probe design.

Country of origin	Sequences	Omitted	No deletions	ΔH69/ΔV70 only	ΔY144 only	ΔH69/ΔV70 and ΔY144
**Australia**	21	0	20 (95.2 %)	0	0	1 (4.8 %)
**Denmark**	107	0	99 (92.5 %)	8 (7.5 %)	0	0
**UK**	965	5	725 (75.5 %)	8 (0.8 %)	0	227 (23.7 %)
**New Zealand**	13	0	13	0	0	0
**Sweden**	2	0	2	0	0	0
**Thailand**	3	0	3	0	0	0
**USA**	25	0	25	0	0	0
**Total**	1136	5	887	16	0	228

Supplementary Table S2. Oligonucleotide primers and probes for common SARS-CoV-2 S gene and B.1.1.7 primer/probe sets.

Oligonucleotide	Sequence	Tm (ºC)	Secondary structure potential (kcal/mol)
Forward primers (F1-F4) targeting all SARS-CoV-2 variants			
SARS-CoV-2 S gene - **F1**	TCT**t**T**t**CCAATGTTACTTGGTTC	54.3	-1.52
SARS-CoV-2 S gene - **F2**	TCT**t**T**t**CCAATGTTACTTGGT**t**C	55.9	-1.52
SARS-CoV-2 S gene - **F3**	T**t**ACCT**t**TCT**t**TTCCAATGTTAC C	54.5	1.51
SARS-CoV-2 S gene - **F4**	CT**t**ACCT**t**TCT**t**TTCCAATGT**t**AC	56.4	1.51
Detection probes (P1-P4) targeting all SARS-CoV-2 variants			
SARS-CoV-2 S gene - **P1**	AGAGGTTTGATAACCCTGTCCTACCA	59.1	-1.97
SARS-CoV-2 S gene - **P2**	AGAGGTTTGATAACCCTGTCC**t**ACCA	60.3	-1.97
SARS-CoV-2 S gene / B.1.1.7 - **P3**	AGAGGTTTGATAACCCTG**t**CC**t**ACCA	61.9	-1.97
SARS-CoV-2 S gene / B.1.1.7- **P4**	T**t**TGCTTCCACTGAGAAGTC**t**AACAT	59.0	-1.48
Reverse primers (R1-R2) targeting all SARS-CoV-2 variants			
SARS-CoV-2 S gene - **R1**	AGTAGGGACTGGGTCTTCGAATCT	58.9	-0.94
SARS-CoV-2 S gene - **R2**	GTAGGGACTGGGTCTTCGAATCTA	57.3	-0.94
Forward primers (F1-F4) targeting the 1st deletion (ΔH69/ΔV70)			
B.1.1.7 - **F1**	GTTACTTGGTTCCATGCTATCTCTG	55.3	1.11
B.1.1.7 - **F2**	GT**t**ACTTGGTTCCATGCTATCTCT	56.3	1.11
B.1.1.7 - **F3**	GT**t**ACT**t**GGTTCCATGCTATCTC	56.8	1.11
B.1.1.7 - **F4**	GTTCCATGCTATCTCTGGGACC	57.1	-0.62
Detection probes (P1-P4) targeting the 1st deletion (1)			
B.1.1.7 - **P1**	ATGCTATCTCTGGGACCAATGGTACT	59.1	-0.96
B.1.1.7 - **P2**	ATGCTATCTCTGGGACCAATGG**t**ACT	60.9	-0.96
B.1.1.7 - **P3**	TGCTATCTCTGGGACCAATGGTACT	59.1	-0.96
B.1.1.7 - **P4**	TGCTATCTCTGGGACCAATGG**t**ACT	61.0	-0.96
Reverse primers (R1-R37) targeting the 2nd deletion (ΔY144)			
B.1.1.7 - **R1**	T**t**TGTTGTTTTTGTGGTAA**a**CACC	55.2	-1.51
B.1.1.7 - **R2**	T**t**GTTGTT**t**TTGTGGTAA**a**CACC	56.9	-1.51
B.1.1.7 - **R3**	TGTTGTT**t**TTGTGGTAA**a**CACCC	57.1	-1.51
B.1.1.7 - **R4**	GTTGTT**t**TTGTGGTAA**a**CACCC	55.6	-1.51

B.1.1.7 - **R5**	TtGTtGTTtTTGTGGTAAaCAC	56.6	-1.51
B.1.1.7 - **R6**	TGtTGtTTTtGTGGTAAaCAC	56.1	-1.51
B.1.1.7 - **R7**	CAACtTTTGTTGTTTTTGTGGTAAACAC	55.6	-3.34
B.1.1.7 - **R8**	CAACtTtTGTTGTTTTTGTGGTAAACAC	56.7	-3.34
B.1.1.7 - **R9**	CAaCtTTTGTTGTTTTTGTGGTAAACAC	56.6	-3.34
B.1.1.7 - **R10**	CAACtTtTGTTGTTTTTGTGGTAAACA	56.0	-2.38
B.1.1.7 - **R11**	CAaCtTtTGTTGTTTTTGTGGTAAACA	57.0	-2.38
B.1.1.7 - **R12**	CAaCtTtTGTTGTTTTTGTGGTAAAC	55.8	-2.14
B.1.1.7 - **R13**	CAACtTtTGTTGTTTTTGTGGTAAAC	54.7	-2.14
B.1.1.7 - **R14**	CAACtTTtGTTGTTTTTGTGGTAAAC	55.0	-2.14
B.1.1.7 - **R15**	CAACTtTtGTTGTTTTTGTGGTAAAC	55.3	-2.14
B.1.1.7 - **R16**	CAACTTTtGTTGTTTTTGTGGTAAAC	54.1	-2.14
B.1.1.7 - **R17**	CAACTtTTGTTGTTTTTGTGGTAAAC	53.8	-2.14
B.1.1.7 - **R18**	CAACtTTTGTTGTTTTTGTGGTAAAC	53.6	-2.14
B.1.1.7 - **R19**	CAACTTTtGTTGtTTTTGTGGTAAAC	55.4	-2.14
B.1.1.7 - **R20**	CAACTtTTGTTGtTTTTGTGGTAAAC	55.1	-2.14
B.1.1.7 - **R21**	CAACTtTtGTTGTTTTTGTGGTAAA	54.4	-2.14
B.1.1.7 - **R22**	CAACTTTtGTTGtTTTTGTGGTAAA	54.5	-2.14
B.1.1.7 - **R23**	CAACtTTtGTTGtTTTTGTGGTAAAC	56.3	-2.14
B.1.1.7 - **R24**	CAACTtTtGTTGtTTTTGTGGTAAAC	56.6	-2.14
B.1.1.7 - **R25**	CAACtTTtGTTGTTTTTGTGGTAAACA	56.2	-2.38
B.1.1.7 - **R26**	CAACTtTtGTTGTTTTTGTGGTAAACA	56.5	-2.38
B.1.1.7 - **R27**	CAACTTTtGTTGtTTTTGTGGTAAACA	56.6	-2.38
B.1.1.7 - **R28**	CAACtTTtGTTGTTTTTGTGGTAACC	56.5	-2.26
B.1.1.7 - **R29**	CAACtTTtGTTGTTTTTGTGGTAAGC	56.7	-2.26
B.1.1.7 - **R30**	CAACTtTtGTTGTTTTTGTGGTAACC	56.9	-2.26
B.1.1.7 - **R31**	CAACTtTtGTTGTTTTTGTGGTAAGC	57.1	-2.26
B.1.1.7 - **R32**	CAACTtTtGTTGTTTTTGTGGTACAC	56.8	-2.26
B.1.1.7 - **R33**	CAACtTTtGTTGtTTTTGTGGTAAGC	58.0	-2.26
B.1.1.7 - **R34**	CAACtTTtGTTGTTtTTGTGGTAAGC	58.0	-2.26
B.1.1.7 - **R35**	CAACtTTtGTTGTTTTtGTGGTAAGC	58.2	-2.26
B.1.1.7 - **R36**	CAACtTTtGTTGTTTTTGtGGTAAGC	58.2	-2.26
B.1.1.7 - **R37**	CAACtTTtGTTGTTTTTGTGGtAAGC	58.4	-2.26

- Primers/probes highlighted in green comprise the final lineage B.1.1.7 S gene primer/probe set
- Nucleotides in lowercase and bold denote LNA-modified bases
- Nucleotides in red font indicate mismatch bases used for SNP detection
- F, forward primer; P, probe; R, reverse primer

Supplementary Table S3. Assessment of clinical performance according to QUADUS-2.

Item	Question	Answer	Domain	Risk of bias
**1.**	Was a consecutive or random sample of patients enrolled?	Omitted - Not applicable	Patient selection	Low
**2.**	Was a case-control design avoided?	Yes		
**3.**	Did the study avoid inappropriate exclusions?	Yes		
**4.**	**Applicability:** Are there concerns that the included patients do not match the review question?	Low		
**5.**	Were the index test results interpreted without knowledge of the results of the reference standard?	Omitted - Not applicable	Index test	Low
**6.**	If a threshold was used, was it prespecified?	Yes		
**7.**	**Applicability:** Are there concerns that the index test, its conduct, or its interpretation differ from the review question?	Low		
**8.**	Is the reference standard likely to correctly classify the target condition?	Yes	Reference standard	Low
**9.**	Were the reference standard results interpreted without knowledge of the results of the index test results?	Yes		
**10.**	**Applicability:** Are there concerns that the target condition as defined by the reference standard does not match the review question?	No		
**11.**	Was there an appropriate interval between index test(s) and reference standard?	Yes	Flow and timing	Low
**12.**	Did all patients receive a reference standard?	Yes		
**13.**	Did all patients receive the same reference standard?	Unclear		
**14.**	Were all patients included in the analysis?	Yes		

Supplementary Table S4. Interpretation of SARS-CoV-2 test results and corresponding actions.

SARS-CoV-2 S gene	ΔCt between B.1.1.7 and SARS-CoV-2 S gene[a]	Human RNase P	Result interpretation	Report
+	Max 5 Ct	+/ND	SARS-CoV-2 B.1.1.7 detected	SARS-CoV-2 B.1.1.7 positive
+	Min 8 Ct	+/ND	SARS-CoV-2 ΔH69/ΔV70 deletion detected	SARS-CoV-2 B.1.1.7 negative
+	Min 20 Ct	+/ND	Other lineage of SARS-CoV-2 detected	SARS-CoV-2 B.1.1.7 negative
+	ND	+/ND	Consensus or other lineage of SARS-CoV-2 detected	SARS-CoV-2 B.1.1.7 negative
ND	+	+/ND	Inconclusive result	Repeat test on same RNA and/or repeat test on re-extracted RNA. If repeat test remains inconclusive, then report as inconclusive
ND	ND	ND	Invalid result	Invalid

[a] ΔCt = B.1.1.7 assay Ct − SARS-CoV-2 S gene assay Ct
ND, not detected

Supplementary Table S5. Reporting checklist according to STARD guidelines.

Section & Topic	No	Item	Reported on page #
**TITLE OR ABSTRACT**			
	**1**	Identification as a study of diagnostic accuracy using at least one measure of accuracy (such as sensitivity, specificity, predictive values, or AUC)	Yes, abstract: Pg. 4
**ABSTRACT**			
	**2**	Structured summary of study design, methods, results, and conclusions (for specific guidance, see STARD for Abstracts)	Pg. 4
**INTRODUCTION**			
	**3**	Scientific and clinical background, including the intended use and clinical role of the index test	Pg. 5-6
	**4**	Study objectives and hypotheses	Pg. 5-6
**METHODS**			
Study design	**5**	Whether data collection was planned before the index test and reference standard were performed (prospective study) or after (retrospective study)	Pg. 21-23
Participants	**6**	Eligibility criteria	Pg. 21-23
	**7**	On what basis potentially eligible participants were identified (such as symptoms, results from previous tests, inclusion in registry)	Previous SARS-CoV-2 positive test Pg. 21-23
	**8**	Where and when potentially eligible participants were identified (setting, location and dates)	Pg. 21
	**9**	Whether participants formed a consecutive, random or convenience series	Pg. 21-23
Test methods	**10a**	Index test, in sufficient detail to allow replication	Pg. 19-20
	**10b**	Reference standard, in sufficient detail to allow replication	Pg. 19-20
	**11**	Rationale for choosing the reference standard (if alternatives exist)	Pg. 19-20
	**12a**	Definition of and rationale for test positivity cut-offs or result categories of the index test, distinguishing pre-specified from exploratory	Pg. 21-23 **Fig. 2b, c Supplementary Table S4**
	**12b**	Definition of and rationale for test positivity cut-offs or result categories of the reference standard, distinguishing pre-specified from exploratory	Yes, see inclusion criteria – positive for SARS-CoV-2 and sequencing result Pg. 21

	**13a**	Whether clinical information and reference standard results were available<br>to the performers/readers of the index test	Sequencing results not available<br>Pg. 20
	**13b**	Whether clinical information and index test results were available<br>to the assessors of the reference standard	Not available<br>Pg. 22
Analysis	**14**	Methods for estimating or comparing measures of diagnostic accuracy	Pg. 22
	**15**	How indeterminate index test or reference standard results were handled	**Supplementary Fig. S4**<br>**Supplementary Table S4**
	**16**	How missing data on the index test and reference standard were handled	Not applicable
	**17**	Any analyses of variability in diagnostic accuracy, distinguishing pre-specified from exploratory	Pre-specified<br>Pg. 22<br>Pg. 9-10, **Table 3**
	**18**	Intended sample size and how it was determined	Pg. 21-22
**RESULTS**			
Participants	**19**	Flow of participants, using a diagram	Pg. 9<br>**Supplementary Table S4**
	**20**	Baseline demographic and clinical characteristics of participants	Not applicable
	**21a**	Distribution of severity of disease in those with the target condition	Not applicable
	**21b**	Distribution of alternative diagnoses in those without the target condition	Not applicable
	**22**	Time interval and any clinical interventions between index test and reference standard	Pg. 22
Test results	**23**	Cross tabulation of the index test results (or their distribution)<br>by the results of the reference standard	**Fig. 2b**<br>Pg. 9-10, **Table 3**<br>**Supplementary Table S7**
	**24**	Estimates of diagnostic accuracy and their precision (such as 95% confidence intervals)	Pg. 9-10, **Table 3**
	**25**	Any adverse events from performing the index test or the reference standard	None, not applicable
**DISCUSSION**			
	**26**	Study limitations, including sources of potential bias, statistical uncertainty, and generalisability	Pg. 22, QUADAS-2 assessment<br>**Supplementary Table S3**<br>Pg. 9-10, **Table 3**
	**27**	Implications for practice, including the intended use and clinical role of the index test	Pg. 4, Abstract

			Pg. 11-17, Discussion
**OTHER INFORMATION**			
	**28**	Registration number and name of registry	Not applicable
	**29**	Where the full study protocol can be accessed	Not applicable
	**30**	Sources of funding and other support; role of funders	Pg. 30, Funding statement

Supplementary Table S6. Cross-reactivity (specificity) testing.

Organism	Vircell Cat. No.	SARS-CoV-2 S gene	B.1.1.7
**HCoV-OC43**	MBC135-R	0/3	0/3
**CoV**	MBC090	0/3	0/3
**SARS-CoV (2003)**	MBC136-R	0/3	0/3
**MERS-CoV**	MBC132	0/3	0/3
**Influenza A H1N1 (Brisbane/59/2007)**	MBC028	0/3	0/3
**Novel Influenza A H1N1 (California/07/2009)**	MBC082	0/3	0/3
**Influenza A H3N2 (Perth/16/2009)**	MBC029	0/3	0/3
**Influenza A H5N1 (Viet Nam/1194/2004 x Puerto Rico/8/1934)**	MBC052	0/3	0/3
**Influenza B (/Brisbane/60/2008)**	MBC030	0/3	0/3
**Human parainfluenza 1**	MBC105	0/3	0/3
**Respiratory syncytial virus (subtype A)**	MBC041	0/3	0/3
**Human rhinovirus (B14)**	MBC091	0/3	0/3
**SARS-CoV-2 positive control**	—	3/3	3/3
**Negative control**	—	0/3	0/3

Supplementary Table S7. Overview of clinical sample RT-qPCR results, lineage, and GISAID information.

rTEST COVID-19 qPCR B.1.1.7 kit				Sequencing		
Sample ID	B.1.1.7 PCR [Ct]	S gene PCR [Ct]	ΔCt	Sequencing outcome (GISAID lineage)	Name in GISAID	Accession ID in GISAID
1	23.8	23.6	0.2	B.1.1.7	UKBA-706	EPI_ISL_875525
2	18.0	18.1	−0.1	B.1.1.7	UKBA-707	EPI_ISL_875526
3	33.9	34.5	−0.5	B.1.1.7	UKBA-801	EPI_ISL_831667
4	36.4	36.0	0.5	B.1.1.7	UKBA-802	EPI_ISL_831668
5	32.3	31.5	0.8	B.1.1.7	UKBA-708	EPI_ISL_875527
6	37.5	35.2	2.3	B.1.1.7	UKBA-803	EPI_ISL_831672
7	23.7	25.0	−1.3	B.1.1.7	UKBA-714	EPI_ISL_875521
8	25.3	24.8	0.5	B.1.1.7	UKBA-713	EPI_ISL_875520
9	No Ct	30.6	-	B.1.160	UKBA-701	EPI_ISL_875530
10	28.8	28.5	0.3	B.1.1.7	UKBA-703	EPI_ISL_875522
11	32.0	26.9	5.0	B.1.1.7	UKBA-705	EPI_ISL_875524
12	25.2	22.9	2.3	B.1.1.7	UKBA-704	EPI_ISL_875523
13	32.2	24.5	7.7	B.1.258	UKBA-702	EPI_ISL_875528
14	No Ct	28.8	-	B.1.1.243	UKBA-715	EPI_ISL_875516
15	No Ct	29.6	-	B.1.177	UKBA-716	EPI_ISL_875533
16	No Ct	24.4	-	B.1.177	UKBA-717	EPI_ISL_875534
17	34.3	29.6	4.7	B.1.1.7	UKBA-718	EPI_ISL_875517
18	30.8	30.2	0.6	B.1.1.7	UKBA-719	EPI_ISL_875518
19	25.2	22.6	2.6	B.1.1.7	UKBA-720	EPI_ISL_875519
20	23.2	13.2	10.1	B.1.258	UKBA-722	EPI_ISL_875529
21	No Ct	12.7	-	B.1.1.170	UKBA-723	EPI_ISL_875532
22	No Ct	25.8	-	B.1.1.170	UKBA-724	EPI_ISL_875538
23	21.1	18.8	2.3	B.1.1.7	UKBA-804	EPI_ISL_831669
24	29.5	26.9	2.6	B.1.1.7	UKBA-805	EPI_ISL_831670
25	22.8	20.8	1.9	B.1.1.7	UKBA-806	EPI_ISL_831673
26	26.2	24.2	2.0	B.1.1.7	UKBA-807	EPI_ISL_831671
27	21.3	18.6	2.6	B.1.1.7	UKBA-808	EPI_ISL_831674
28	26.7	15.2	11.5	B.1.258	UKBA-809	EPI_ISL_831676
29	30.0	27.8	2.3	B.1.1.7	UKBA-814	EPI_ISL_831675
30	23.5	21.8	1.6	B.1.1.7	UKBA-815	EPI_ISL_831663
31	22.9	18.7	4.1	B.1.1.7	UKBA-816	EPI_ISL_831664
32	28.5	24.2	4.3	B.1.1.7	UKBA-817	EPI_ISL_831665
33	No Ct	30.1	-	B.1.258	UKBA-818	EPI_ISL_831666
34	25.4	24.0	1.4	B.1.1.7	UKBA-501	EPI_ISL_779651
35	29.4	29.1	0.3	B.1.1.7	UKBA-502	EPI_ISL_779652

36	30.7	28.9	1.8	B.1.1.7	UKBA-503	EPI_ISL_779653
37	31.5	30.0	1.5	B.1.1.7	UKBA-504	EPI_ISL_779654
38	34.3	29.9	4.4	B.1.1.7	UKBA-505	EPI_ISL_779655
39	31.5	30.8	0.7	B.1.1.7	UKBA-506	EPI_ISL_779656
40	37.9	32.2	5.7	B.1.1.7	UKBA-507	EPI_ISL_779657
41	30.2	29.1	1.1	B.1.1.7	UKBA-508	EPI_ISL_779658
42	37.6	34.4	3.2	B.1.1.7	UKBA-509	EPI_ISL_779659
43	39.7	35.2	4.5	B.1.1.7	UKBA-512	EPI_ISL_779660
44	25.8	14.7	11.1	B.1.258	UKBA-1001	EPI_ISL_903980
45	No Ct	23.1	-	B.1.1.170	UKBA-1002	EPI_ISL_903981
46	24.4	15.2	9.2	B.1.258	UKBA-1004	EPI_ISL_903983
47*	35.8	27.1	8.7	B.1.258	UKBA-1005	EPI_ISL_903984
48*	29.1	18.3	10.8	B.1.258	UKBA-1006	EPI_ISL_903985
49	15.6	16.8	−1.2	B.1.1.7	UKBA-1007	EPI_ISL_903986
50	25.4	16.1	9.3	B.1.258	UKBA-1008	EPI_ISL_903987
51	No Ct	16.5	-	B.1.160	UKBA-1009	EPI_ISL_903988
52	19.9	17.4	2.5	B.1.1.7	UKBA-1010	EPI_ISL_903989
53	27.6	25.5	2.1	B.1.1.7	UKBA-1011	EPI_ISL_903990
54*	37.1	26.0	11.1	B.1.258	UKBA-1012	EPI_ISL_903991
55	No Ct	15.1	-	B.1.177	UKBA-1013	EPI_ISL_903992
56	28.0	19.6	8.4	B.1.258	UKBA-1014	EPI_ISL_903993
57	No Ct	23.4	-	B.1.1.277	UKBA-1015	EPI_ISL_903994
58*	35.5	26.9	8.6	B.1.258	UKBA-1016	EPI_ISL_903995
59	No Ct	16.3	-	B.1.160	UKBA-1017	EPI_ISL_903996
60	33.2	22.9	10.3	B.1.258	UKBA-1018	EPI_ISL_903997
61	26.5	17.5	9.0	B.1.258	UKBA-1020	EPI_ISL_903999
62	19.8	20.7	−0.9	B.1.1.7	UKBA-1021	EPI_ISL_904000
63	No Ct	18.9	-	B.1.221	UKBA-1022	EPI_ISL_904001
64*	35.2	25.3	9.9	B.1.258	UKBA-1023	EPI_ISL_904002
65*	No Ct	28.9	-	B.1.258	UKBA-1024	EPI_ISL_904003
66	21.7	19.4	2.4	B.1.1.7	UKBA-1101	EPI_ISL_959643
67	No Ct	17.1	-	B.1.160	UKBA-1102	EPI_ISL_959642
68	25.8	16.4	9.4	B.1.258	UKBA-1103	EPI_ISL_959648
69	17.7	15.3	2.4	B.1.1.7	UKBA-1104	EPI_ISL_959645
70	No Ct	17.9	-	B.1.1.170	UKBA-1105	EPI_ISL_959647
71	21.7	18.9	2.9	B.1.1.7	UKBA-1106	EPI_ISL_959646
72	26.5	22.6	3.9	B.1.1.7	UKBA-1107	EPI_ISL_959644
73	32.3	20.6	11.6	B.1.258	UKBA-1108	EPI_ISL_959649
74	No Ct	25.0	-	B.1.1.7	UKBA-1109	EPI_ISL_959637
75*	33.8	30.3	3.5	B.1.1.7	UKBA-1110	EPI_ISL_959638
76*	21.0	19.5	1.5	B.1.1.7	UKBA-1111	EPI_ISL_959639

77*	26.3	23.8	2.5	B.1.1.7	UKBA-1112	EPI_ISL_959640
78	No Ct	23.2	-	B.1.1.170	UKBA-1113	EPI_ISL_959641
79	29.9	27.5	2.5	B.1.1.7	UKBA-1114	EPI_ISL_959627
80	32.7	24.2	8.5	B.1.258	UKBA-1115	EPI_ISL_959630
81	31.0	28.8	2.1	B.1.1.7	UKBA-1116	EPI_ISL_959628
82	25.3	23.0	2.3	B.1.1.7	UKBA-1117	EPI_ISL_959626
83	26.9	16.7	10.2	B.1.258	UKBA-1118	EPI_ISL_959631
84	20.9	17.5	3.4	B.1.1.7	UKBA-1119	EPI_ISL_959629
85	23.8	19.0	4.8	B.1.1.7	UKBA-1120	EPI_ISL_959632
86	21.6	19.3	2.2	B.1.1.7	UKBA-1121	EPI_ISL_959633
87	19.0	16.3	2.7	B.1.1.7	UKBA-1122	EPI_ISL_959634
88	19.4	17.2	2.1	B.1.1.7	UKBA-1123	EPI_ISL_959635
89	28.0	16.7	11.4	B.1.258	UKBA-1124	EPI_ISL_959636
90	18.1	16.7	1.4	B.1.1.7	UKBA-1207	EPI_ISL_959604
91	26.4	22.8	3.6	B.1.1.7	UKBA-1208	EPI_ISL_959605
92	15.5	13.3	2.3	B.1.1.7	UKBA-1209	EPI_ISL_959606
93	23.8	14.1	9.7	B.1.258	UKBA-1210	EPI_ISL_959607
94	18.4	16.9	1.5	B.1.1.7	UKBA-1211	EPI_ISL_959608
95	17.2	15.3	1.8	B.1.1.7	UKBA-1212	EPI_ISL_959609
96	22.8	21.5	1.3	B.1.1.7	UKBA-1213	EPI_ISL_959610
97	16.4	14.4	2.0	B.1.1.7	UKBA-1214	EPI_ISL_959611
98	18.9	16.9	2.1	B.1.1.7	UKBA-1215	EPI_ISL_959612
99	14.9	13.4	1.5	B.1.1.7	UKBA-1216	EPI_ISL_959613
100	16.1	14.4	1.8	B.1.1.7	UKBA-1217	EPI_ISL_959614
101	24.3	22.0	2.3	B.1.1.7	UKBA-1218	EPI_ISL_959615
102	17.6	15.3	2.3	B.1.1.7	UKBA-1219	EPI_ISL_959616
103	20.2	17.8	2.4	B.1.1.7	UKBA-1221	EPI_ISL_959617
104	15.9	14.1	1.8	B.1.1.7	UKBA-1222	EPI_ISL_959618
105	No Ct	23.9	-	B.1.258	UKBA-1223	EPI_ISL_959619
106	27.1	16.9	10.2	B.1.258	UKBA-1224	EPI_ISL_959620

* Samples with an asterisk were re-tested due to an inconclusive result in the first test. The results depict the re-test values.

Supplementary Table S8. Surveillance of lineage B.1.1.7 prevalence in the Slovak Republic.

Region	B.1.1.7 (ΔH69/ΔV70 + ΔY144)	ΔH69/ΔV70 only	Other lineage of SARS-CoV-2	Inconclusive	Sample size	B.1.1.7 prevalence
Retest date: 2 February 2021						
**Banská Bystrica**	132	1	46	4	183	74%
**Bratislava**	306	12	73	4	395	78%
**Košice**	163	0	65	12	240	71%
**Nitra**	144	5	51	5	205	72%
**Prešov**	124	3	66	3	196	64%
**Trenčin**	131	5	25	6	167	81%
**Trnava**	357	6	57	17	437	85%
**Žilina**	69	9	54	7	139	52%
**Total**	**1426**	**41**	**437**	**58**	**1962**	**75%**
Retest date: 17 February 2021						
**Banská Bystrica**	242	0	156	4	402	61%
**Bratislava**	217	0	46	4	267	83%
**Košice**	235	1	114	18	368	67%
**Nitra**	101	0	45	5	151	69%
**Prešov**	145	3	45	27	220	75%
**Trenčin**	248	0	50	0	298	83%
**Trnava**	348	0	53	17	418	87%
**Žilina**	174	0	84	0	258	67%
**Total**	**1710**	**4**	**593**	**75**	**2382**	**74%**
Retest date: 3 March 2021						
**Banská Bystrica**	211	0	39	16	266	84%
**Bratislava**	222	1	22	11	256	91%
**Košice**	225	0	82	60	367	73%
**Nitra**	128	2	21	16	167	85%
**Prešov**	206	4	54	44	308	78%
**Trenčin**	322	7	51	32	412	85%
**Trnava**	349	6	35	19	409	89%
**Žilina**	262	1	90	4	357	74%
**Total**	**1925**	**21**	**394**	**202**	**2542**	**82%**
